# High-Efficiency Solar Hybrid Photovoltaic/Thermal System Enabled by Ultrathin Asymmetric Fabry–Perot Cavity

**DOI:** 10.1021/acsphotonics.4c01315

**Published:** 2025-02-06

**Authors:** Ran Wei, Tianshu Xu, Chunlei Guo

**Affiliations:** The Institute of Optics, University of Rochester, Rochester, New York 14627, United States

**Keywords:** solar hybrid photovoltaic/thermal system, asymmetric metal-dielectric-metal optical coating, quad-band spectrum splitter

## Abstract

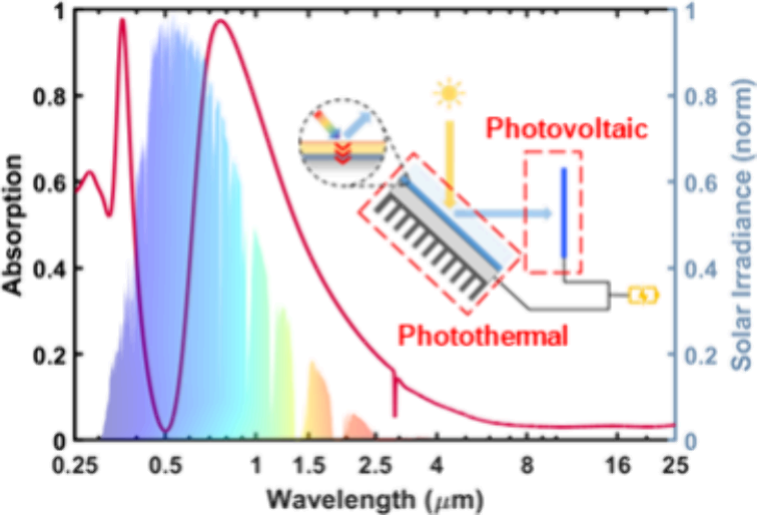

Solar hybrid photovoltaic/thermal (HPT) systems maximize the overall solar energy conversion by simultaneously converting solar energy into electrical and thermal energy. However, the practical implementation of HPT systems is hindered by a lack of suitable optical materials capable of efficiently splitting the incident solar spectrum into the desired photovoltaic (PV) and photothermal (PT) bands. In this work, we provide the first demonstration of a multifunctional asymmetric metal-dielectric-metal (asym-MDM) optical coating to be used in an HPT system. The asym-MDM serves as the dual function of a quad-band spectrum splitter and a thermal receiver, leveraging on the multiorder spectral responses and the lossy nature of nickel. Moreover, silica aerogel is employed as a transparent insulting material to enhance the thermal storage capability, while the heat is effectively utilized for increasing the temperature difference of a thermoelectric generator (TEG). As a result, a simple and highly compact HPT system is developed, with simultaneous extraordinary heat mitigation of the single-junction amorphous silicon solar cell and heat generation at the hot side of the TEG. This leads to 63.9 and 370% performance improvements for the PV and PT subsystems at a solar concentration of 3, respectively. Asym-MDM will provide a low-cost yet high-efficiency solution for application of an HPT system in solar energy harnessing.

## 1 Introduction

Solar energy is a nearly inexhaustible energy source and a solution to addressing global energy demands. Currently, two types of major technologies have been developed to harness solar energy: the photovoltaic (PV) process that directly converts sunlight into usable electricity^1,2^ and the photothermal (PT) process that converts sunlight into heat for solar-thermal applications.^3,4^ These two technologies can provide solutions in meeting the ever-growing energy demands while minimizing environmental impact.

In a typical PV system that utilizes single-junction PV cells (e.g., amorphous silicon PV cells), only photon energy around the bandgap of the PV cell can be converted into electricity, while the remaining photon energy will be absorbed and converted to heat within the PV cell. The heat conversion limits the solar conversion maximum theoretical efficiency, while the generated heat reduces the PV cell efficiency due to increased carrier recombination rates and decrease in the cell’s bandgap.^5^ Meanwhile, the heat reduces the PV cell’s lifetime, with the aging rate nearly doubling with every 10 °C increase in temperature.^6^ To overcome these issues, solar hybrid photovoltaic/thermal (HPT) technology has been developed to spectrally separate the solar spectrum into distinct bands to power PV and PT subsystems.^7,8^ This approach utilizes the synergistic advantages of both PV and PT energy generation:^9^ The PV cell selectively receives photons from a specific range around *E*_g_ to mitigate temperature rise, while the remaining energy is converted into heat at low cost for various solar-thermal applications, including steam generation, sanitation, desalination, and thermoelectric generation.^3,10^

To achieve better thermal management, quad-band spectrum-splitting filters have recently been proposed and demonstrated.^11,12^ The approach utilizes distributed Bragg reflector (DBR) mirrors to separate the entire solar spectrum into a PV band, two thermal bands, and an IR band. However, DBR mirrors are typically tens of micrometers thick due to the periodic variation of low and high index materials, making them relatively costly to produce.

On the other hand, Fabry–Perot (FP) cavity, achieved via an ultrathin film optical coating, has garnered considerable attention because it is lithography-free and cost-effective.^13−15^ Typically, an FP cavity is composed of two metallic mirrors separated by an optically transparent dielectric spacer, i.e., a metal-dielectric-metal (MDM) thin film optical coating. The phase accumulated during the propagation of light within the dielectric medium allows constructive and destructive interference at the air–metal interface, resulting in distinct spectral responses. Ultrathin film-based FP cavities have been extensively studied in the field of nanophotonics, offering diverse applications such as structural coloring,^16−19^ gas sensing,^15,20^ and perfect absorbers.^21−23^ However, since MDMs have intrinsic loss from the metal layer, which is often considered as a waste in designing spectrum splitters for HPT systems, their use in renewable solar energy harnessing systems has never been explored.

**Figure 1:**
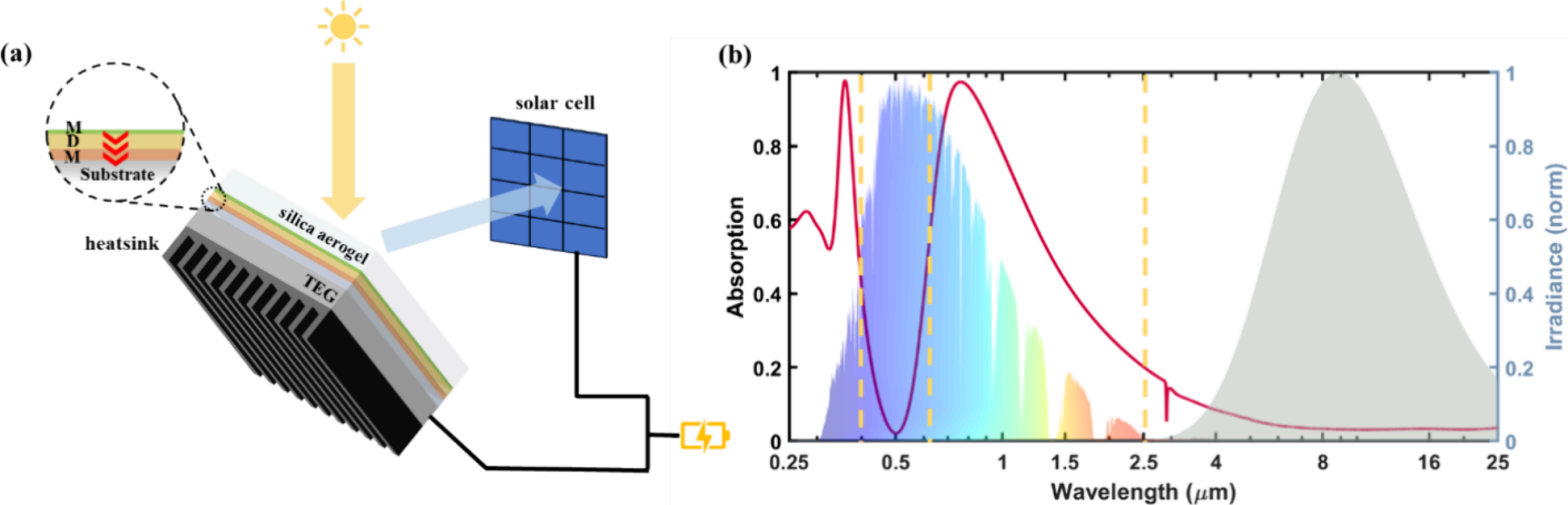
(a) Schematics of the proposed HPT system utilizing an ultrathin asymmetric Fabry–Perot cavity, where it demonstrates (b) a first thermal band exhibiting strong absorption in the UV region, a PV band reflecting off from the surface for an effective photoelectric process, a second thermal band facilitating broadband absorption of the remaining solar spectrum, and an IR band with ultralow emissivity to prevent radiation heat loss. A normalized air mass coefficient (AM) 1.5 solar spectrum and blackbody radiation are plotted together to demonstrate each band’s functionality.

In this work, we present the first demonstration of a multifunctional asymmetric MDM (asym-MDM)-based compact HPT system. The asym-MDM serves as the dual function of a quad-band spectrum splitter and a thermal receiver for enhanced throughput of electricity generation, as depicted in Figure 1a. Rather than avoiding the loss from MDM, we directly transform it into usable heat from our unique spectral design. The asym-MDM leverages the multiorder spectral response of the MDM as well as the lossy nature of its top metal layer, resulting in the formation of four distinct bands: a first thermal band effectively absorbing solar energy in the ultraviolet (UV) region, a PV band aligning with the external quantum efficiency (EQE) of the amorphous Si solar cell, a second broad thermal band covering the remaining solar spectrum, and an IR band exhibiting ultralow emissivity to minimize radiative heat loss (Figure 1b). The quad-band response splits a portion of the solar spectrum to the solar cell, effectively reducing the heat accumulation and thus enabling higher solar concentration to be used. Meanwhile, the thermal bands allow abundant heat to be generated on the asym-MDM for improved thermoelectric generation at the PT module. As a result, our HPT system leads to 63.9 and 370% performance improvements for the PV and PT modules at a solar concentration of 3, respectively.

## 2 Results and Discussion

**Figure 2:**
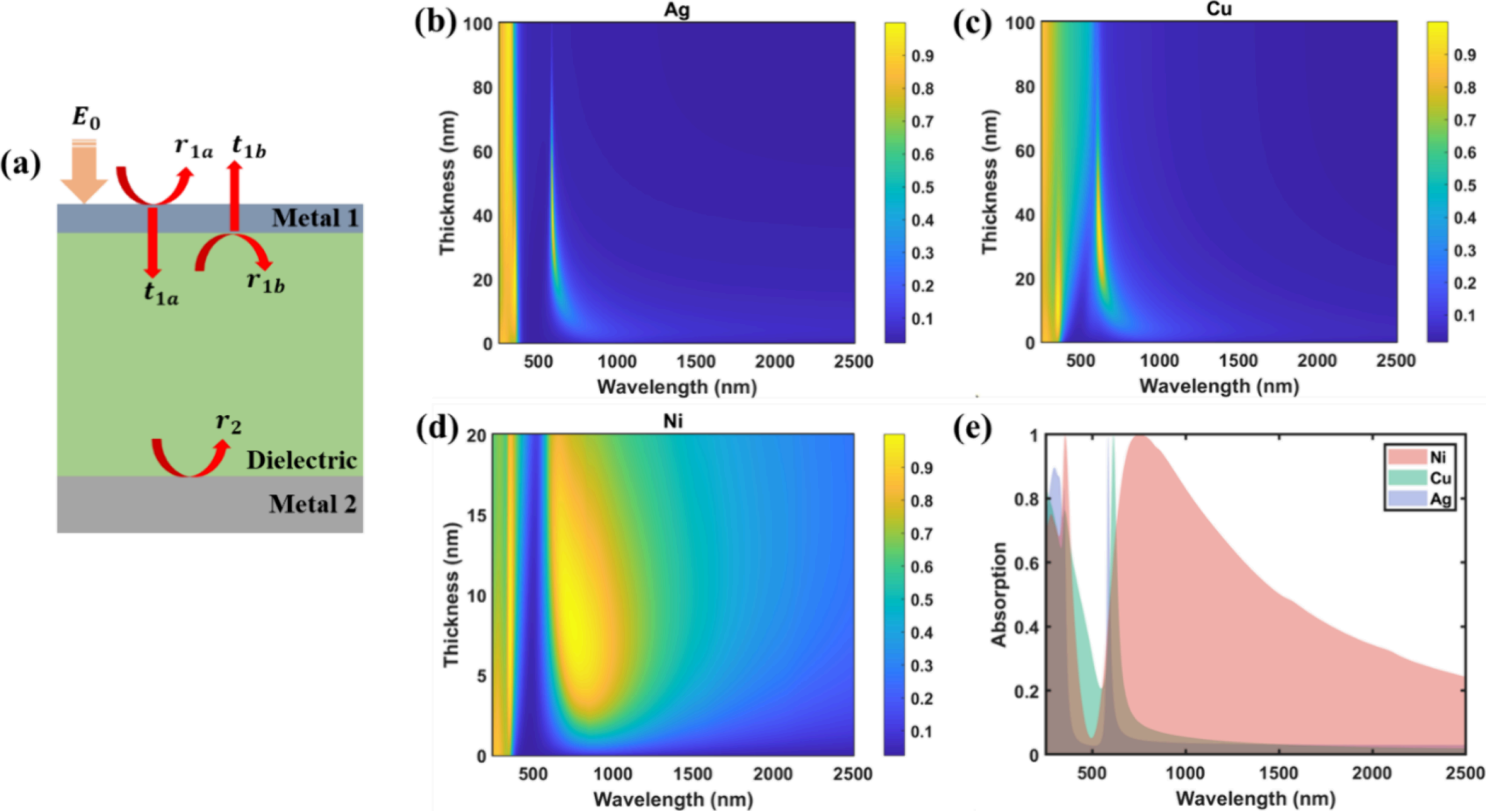
(a) Schematic of the asym-MDM structure. (b–d) Absorptions for three different top metals (metal 1) silver (Ag), copper (Cu), and nickel (Ni), respectively, with respect to different thicknesses. The thicknesses of Ag and Cu are varied in the range of 0–100 nm, while the thickness of Ni is varied in the range of 0–20 nm. For all three calculations, the thicknesses of the remaining two layers are fixed, with the dielectric layer being 73 nm and the bottom metal layer (metal 2) being 100 nm. (e) Optimal spectrum responses for the three top metal layers.

Figure 2a shows a typical asym-MDM coating, with incident light entering from the air to the top thin metal layer (referred to as metal 1 in Figure 2a). Generally, an FP cavity exhibits selective absorption due to the destructive interference between the light reflected at the top layer (i.e., *r*_1a_) and the light reflected within the cavity (i.e., denoted as *r*_D_). The latter can be represented as follows:^24^
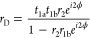
(1)where *r*_1b_ and *r*_2_ represent the reflection coefficient at the dielectric-metal interface for the top and bottom metal layer respectively, *t*_1a_ and *t*_1b_ denote the transmission coefficient at the metal-dielectric and metal-air interface respectively, and ϕ is the single-pass phase accumulation during the propagation in the lossless dielectric (, where *n* and *L* are the refractive index and physical thickness of the dielectric, respectively, and λ is the wavelength of light). Detailed derivation of eq (1) is given in the Methods section. To have a total destructive interference between *r*_1a_ and *r*_D_, a phase difference with an odd number of π is required, i.e., , where *k* = 0, 1, 2, 3..., corresponding to the multiorder absorption peaks from the FP cavity. Moreover, there exists a reflection peak between the two adjacent absorption peaks, as can be seen from Figure 1b, which can be tuned (i.e., by changing the accumulated phase of *r*_D_ as it propagates within the cavity) to overlap with the peak of the external quantum efficiency of the solar cell for efficient photovoltaic conversion. If the reflection peak mismatches with the bandgap, then additional heat will be generated on the solar cell with reduced power output.

In our design, the bottom metal layer (referred to as metal 2 in Figure 2a) is optically opaque by depositing a 100 nm silver (Ag) layer, ensuring zero transmission, whereas TiO_2_ is used as the lossless dielectric layer. The absorption of TiO_2_ in the UV region ensures stronger absorption of the corresponding solar spectrum, while the phonon-assisted absorption in IR^25,26^ is far from the blackbody absorption peak studied here (∼8 μm) and essentially is negligible. For the top metal layer, however, the choice of material is crucial in determining the thermal management capability for an effective photothermal process. The full width at half-maximum (fwhm) of the first-order absorption peak in an FP cavity under normal incidence is given by (assuming reflectance from the bottom metal layer is 1)^24^
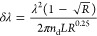
(2)where λ denotes the resonant wavelength of the FP cavity, *R* signifies the reflectance of the top metal layer, and *n*_d_ and *L* represent the refractive index and physical thickness of the lossless dielectric layer, respectively. Accordingly, a top metal layer with a smaller *R* corresponds to a larger fwhm, i.e., a broader absorption response (Figure S1). From Fresnel’s law, metals with the imaginary part of refractive indices significantly larger than the real part (*n* ≪ *k*) exhibit near-unity reflectance, whereas metals with comparable real and imaginary parts of refractive indices (*n* ∼ *k*) have much lower reflectance (see the Supporting Information Note S1). Therefore, the use of the latter kind of metals in an asym-MDM coating as the top layer will lead to a broadened absorption band compared to the former kind, thus enabling an efficient solar-thermal transition process.

To see this clearer, three different metals, i.e., Ag, copper (Cu), and nickel (Ni), are selected as the top metal layer with distinct differences in the real and imaginary parts of their refractive indices, as shown in Figure S2. Their absorption behaviors across the solar spectral range are numerically calculated in Figure 2b–d with varying layer thicknesses. Ag is one of the highly reflective metals commonly employed in mirror coatings, whose imaginary part of the refractive index is much larger than its real part (Figure S2a). This leads to an ultranarrow absorption band in the visible range of only 16.7 nm (Figure 2e). However, the near-unity reflectance covering a substantial portion of the solar spectrum (approximately 800–2500 nm) hinders the solar energy from being transformed into usable heat. Cu is also a metal with strong reflectance, but the difference between its real and imaginary part of refractive index is slightly smaller compared with that of Ag (Figure S2b), thereby exhibiting a nearly 3 times larger absorption band than Ag with an fwhm of 45 nm.

Conversely, Ni has comparable real and imaginary parts of its refractive index (Figure S2c), leading to an ultrabroad absorption band spanning 907.7 nm. This is 54 times larger than that of Ag. The broadband absorption therefore can effectively convert the solar energy into usable heat within the asym-MDM. Additionally, the second-order absorption peak in the ultraviolet (UV) region allows for additional solar absorptance, and the resulting narrow reflection band situated between the first- and second-order absorption peaks can be directed toward the PV cell for highly efficient electricity generation while synergistically mitigating the temperature accumulation on the PV module (see Figure S3 for numerical calculated spectral responses of more metals). The position of the reflection band can be tuned to overlap with the EQE of the PV cell by simply modulating the resonant wavelengths of the two absorption peaks.

**Figure 3:**
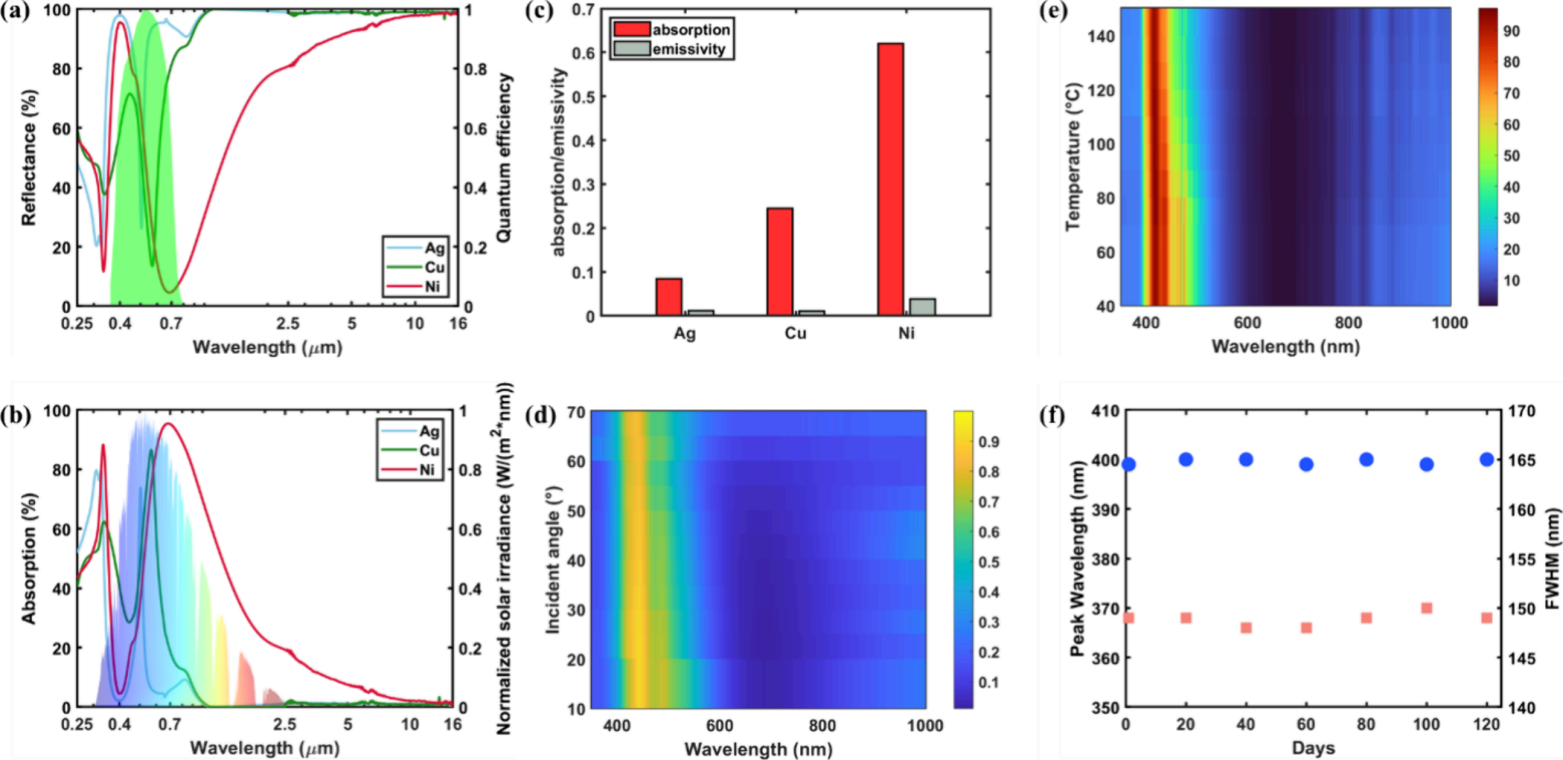
(a) Measured reflectance of the three asym-MDMs, where the shaded green area indicates the quantum efficiency of the amorphous silicon solar cell used in the HPT system. (b) Measured absorptions of the three asym-MDMs. The Ni-based asym-MDM exhibits the broadest absorption band covering a substantial part of the solar spectrum, enabling an efficient photothermal transition process. (c) Calculated average solar absorption and emissivity at *T* = 100 °C for the three asym-MDMs. (d) Measured angular reflectance for Ni-based asym-MDM under p-polarized illumination. (e) Measured reflectance for the Ni-based asym-MDM under different surface temperatures, where negligible change is observed. (f) Peak wavelength and fwhm of the PV band for the Ni-based asym-MDM were monitored in 120 days. The blue dot and pink square represent the peak wavelength and fwhm of the PV band, respectively.

Three asym-MDM coatings are prepared with Ag, Cu, and Ni as the top layer on silica substrates (see the Methods section). The measured spectral responses at normal incidence are shown in Figure 3a,b. Due to fabrication inaccuracy, all three samples exhibit similar blue-shifts from their designs in Figure 2e, causing a slight misalignment with the EQE of the amorphous silicon PV cell (Figure 3a). However, the overall spectral responses for all three asym-MDMs found favorable agreements in terms of spectrum-splitting capability, where the bandwidth of the first-order absorption band witnessed a substantial increment as the top metal layer changed from Ag to Ni (Figure 3b). The Ni-based asym-MDM exhibits the broadest first-order absorption band covering a substantial part of the solar spectrum, which enables efficient thermal energy conversion. For all three asym-MDMs, the narrow reflection band peaks at ∼400 nm.

To quantify the performance of the deposited asym-MDMs, we analyzed the absorption and emissivity for each sample. The spectrally averaged absorption (α̅) is given by the following equation:^27^
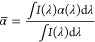
(3)

While the spectrally averaged emissivity (ε̅) is given by the following equation:^28^
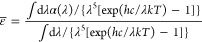
(4)where λ is the wavelength, *I*(λ) is the air mass (AM) 1.5G solar spectrum, α(λ) is the spectral absorption of the asym-MDM, *h* is Planck’s constant, *c* is the speed of light, *k* is the Boltzmann constant, and *T* is the absorber temperature, which is taken as 150 °C (the highest temperature reached in the measurement). Figure 3c summarizes the solar-averaged absorption and blackbody-averaged emissivity for the asym-MDMs. Although Ag-based asym-MDM has the lowest emissivity, indicating minimal thermal radiation loss, it also has a poor solar absorption of less than 10% due to the ultranarrow absorption band. Cu-based asym-MDM has an enhanced solar absorption of 24.5% from the slightly broader absorption band, while Ni-based asym-MDM holds the highest solar absorption of 62% with 3.8% emissivity. As a result, Ni-based asym-MDM exhibited a distinctive spectral response, including a thermal band within the UV region of the solar spectrum, a narrow PV band aligning with the EQE of the amorphous Si solar cell, a wide second thermal band encompassing the broad solar spectrum from 800 to 2500 nm, and an infrared band with low emissivity to minimize radiative heat loss.

Another advantage of asym-MDM is the flexible iridescence control, which can be easily tuned by changing the material of the dielectric layer. Specifically, for the HPT system, low iridescence is required to efficiently reflect the same PV band from concentrated solar. However, in other applications, such as anticounterfeiting, high iridescence is necessary.^29^ Generally, when using high refractive index dielectric material, e.g., TiO_2_, asym-MDM will exhibit low iridescence, vice versa for the dielectric with a low refractive index, e.g., MgF_2_ (see the Supporting Information Note S2). Figure 3d shows the measured angular reflectance under p-polarized illumination (see Figure S4a and Figure S4b for the s-polarized and unpolarized illumination, respectively) for the Ni-based asym-MDM, where the spectral responses show negligible change as the incident angle increases up to 70° (also see Figure S5 for photos taken at different angles for the asym-MDMs).

We further investigate the temperature stability and durability of the asym-MDM, as maintaining a consistent spectral response under varying temperatures and during extended operation is crucial for a stable system output. Figure 3e shows the reflectance for the Ni-based asym-MDM across a wide temperature range from 40 to 150 °C, which has negligible change in both the position and amplitude of the PV and thermal bands. This demonstrates the good temperature stability of the asym-MDM, making it suitable for concentrated solar with elevated temperature. Moreover, we continuously measure the reflectance of the Ni-based asym-MDM over 4 months while it is constantly being tested and stored in ambient condition. Figure 3f presents the corresponding peak position and fwhm of the PV band. Both quantities remain nearly the same after 120 days, demonstrating the exceptional durability of the asym-MDM.

**Figure 4:**
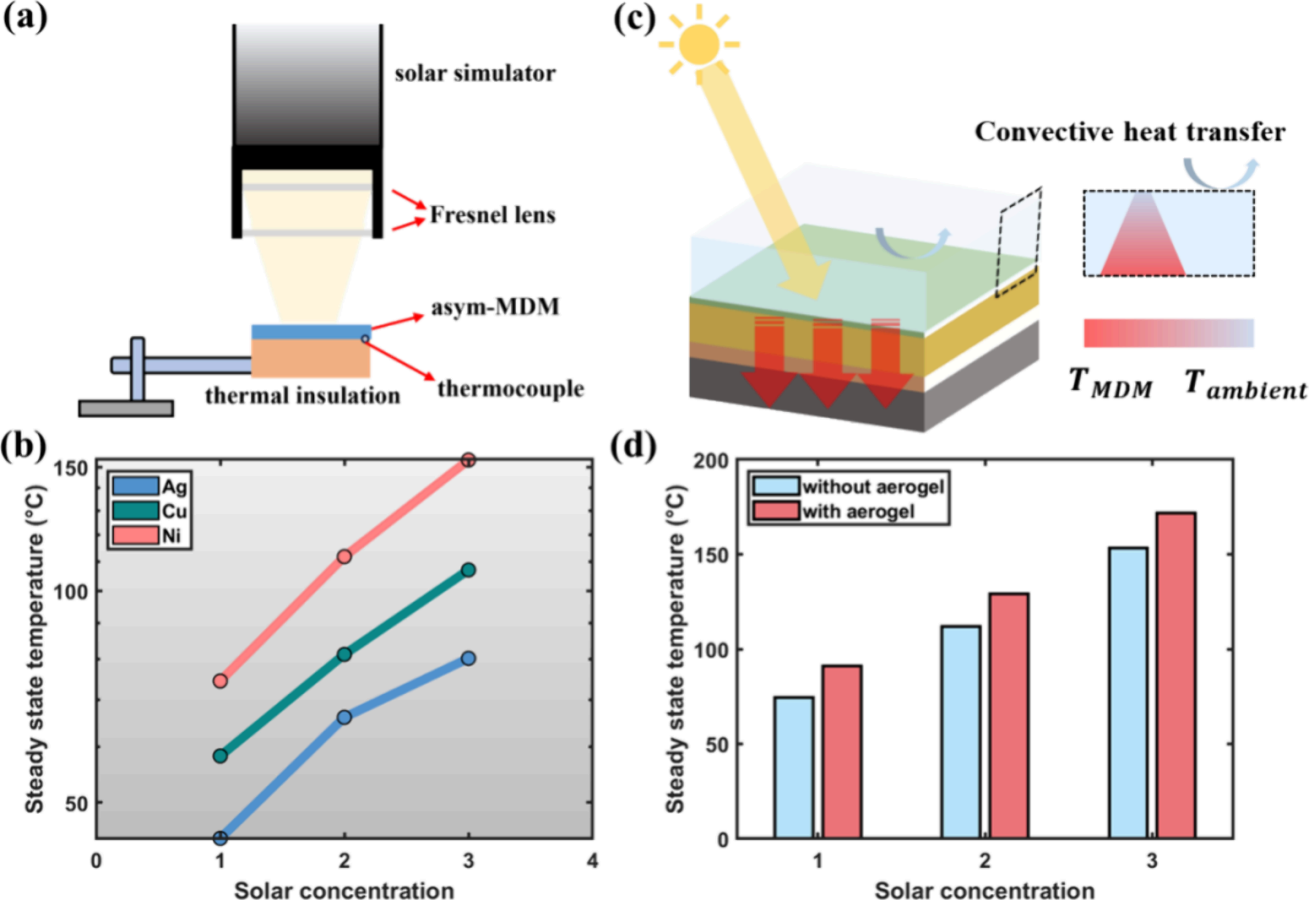
(a) Schematics of the experimental setup for measuring SSTs at different solar concentrations. (b) SSTs of all three asym-MDMs when *C*_opt_ = 1, 2, and 3. (c) Schematics of the silica aerogel-capped asym-MDM, where the convective heat loss can be largely suppressed due to the excellent heat insulation of the silica aerogel while maintaining high transmission for the sunlight. (d) Comparison of SSTs for the Ni-based asym-MDM with and without silica aerogel capping.

To further quantify the superiority of the Ni-based asym-MDM over Ag- and Cu-based asym-MDMs, we measured the steady-state temperatures (SSTs) under different solar concentrations (*C*_opt_). The asym-MDM is placed upon a thermal insulating material to prevent conductive heat exchange with the mounting frame, and the temperature of the substrate’s bottom surface is recorded (Figure 4a). Figure 4b summarizes SSTs for all three asym-MDMs under *C*_opt_ = 1, 2, and 3. As expected, the Ag-based asym-MDM demonstrates the lowest SST and Ni-based asym-MDM has the highest SST. Specifically, when *C*_opt_ = 3, Ni-based asym-MDM reaches an SST of 153.45 °C, which represents a 91.4 and 43.3% increment compared to Ag- and Cu-based asym-MDMs under the same solar concentration, respectively. Therefore, Ni-based asym-MDM provides a much better photothermal conversion capability.

Since the emissivity of the asym-MDM has been largely suppressed, the main factor limiting a higher SST to be reached is the convective heat transfer with the surrounding ambient temperature, which is proportional to the temperature difference Δ*T* between the surface temperature of asym-MDM and ambient temperature. In this regard, we placed a thin layer of silica aerogel atop the asym-MDM structure to achieve elevated SST and thus better thermal storage capability, as depicted in Figure 4c. Silica aerogels are widely recognized as superinsulating materials with ultralow thermal conductivity and high transparency.^30^ Here, we use a commercially available silica aerogel (see Figure S6a,b for its appearance) to cover the top of the Ni-based asym-MDM. The silica aerogel exhibits a solar-weighted average transmittance of 91.4% (Figure S6c). Subsequently, the SSTs for the Ni-based asym-MDM are improved under all solar concentrations, as shown in Figure 4d. An average of ∼18 °C increment is observed from the silica aerogel-capped asym-MDM compared to the bare asym-MDM. This signifies a great potential for this low-cost and straightforward assembly to enhance the overall performance of the HPT system.

**Figure 5:**
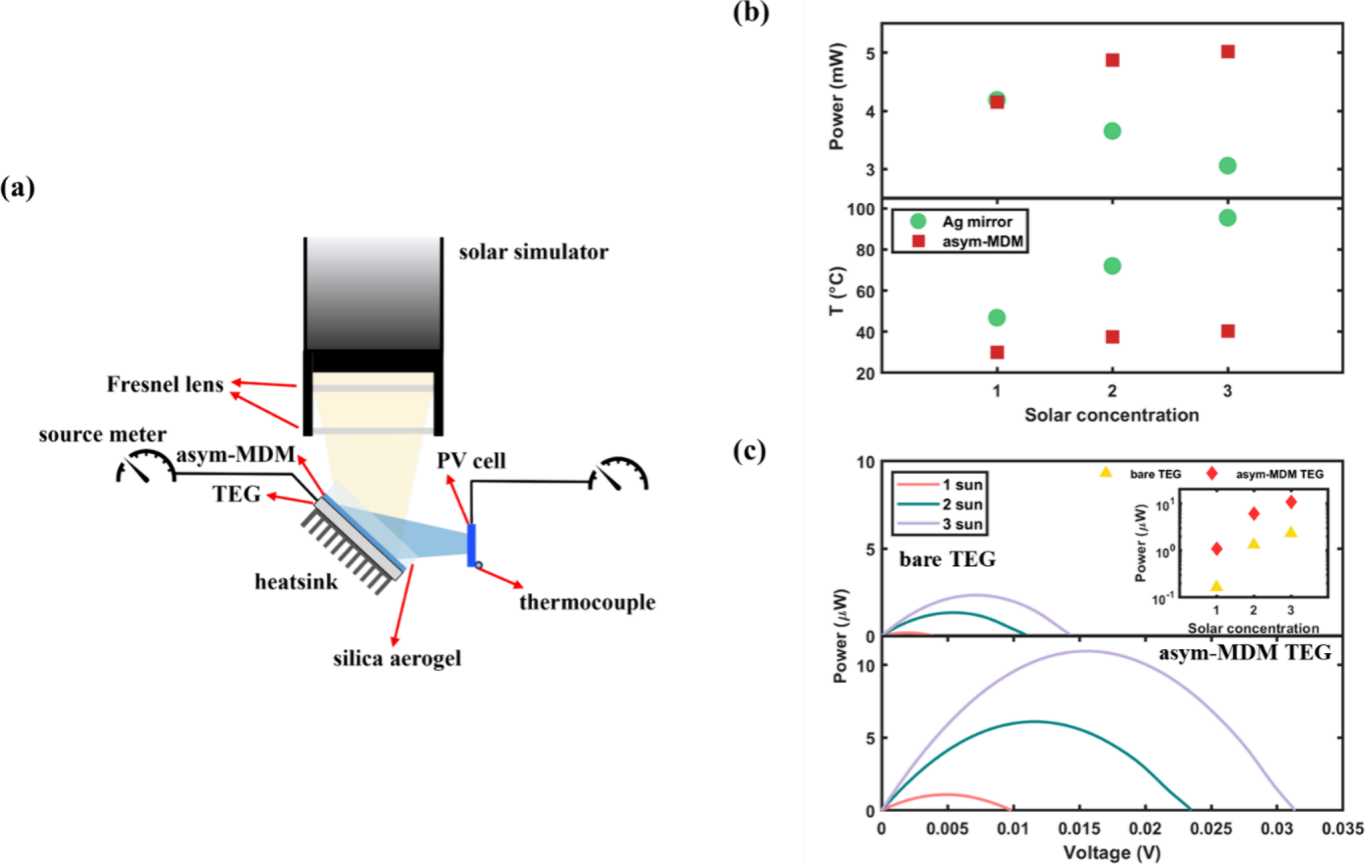
(a) Schematics of the experimentally assembled HPT system using the deposited Ni-based asym-MDM structure. (b) Experimentally measured output power and temperature of the amorphous Si PV cell under different solar concentrations when a Ag mirror and asym-MDM are used, respectively. (c) Comparison of the power–voltage curves between a bare TEG and an asym-MDM attached TEG under different solar concentrations. The inset summarizes the maximum output powers for both conditions.

Finally, we construct a compact HPT system using the dual-functional Ni-based asym-MDM, as schematically shown in Figure 5a (see Figure S7 for the photograph of the experimental setup). The incoming solar light is concentrated by two Fresnel lenses and directed onto the silica aerogel-capped Ni-based asym-MDM. The back side of the asym-MDM is attached to the hot side of a TEG module (2 cm × 2 cm), while a heat sink with the same size is attached to the cold side of the TEG for heat dissipation. The reflected PV band from the asym-MDM is received by a truncated amorphous silicon PV cell (1 cm × 1 cm) for photoelectric generation. The output powers of both the PV cell and TEG are monitored by source meters (Keithley 2450), and the temperature of the PV cell is recorded by a thermocouple attached at the back.

The performance of the Ni-based asym-MDM HPT system is systematically compared to a standalone PV system, where a Ag mirror is used to reflect the entire solar spectrum toward the same PV cell. Figure 5b shows the output power and temperature of the PV cell under different solar concentrations when the Ag mirror and asym-MDM are used, respectively. The temperature of the PV cell experiences a rapid increase in the standalone system, reaching 95.5 °C at *C*_opt_ = 3. Correspondingly, the output power keeps decreasing at higher *C*_opt_, implying performance degradation due to heat accumulation. On contrast, with our asym-MDM HPT system, the temperature of the PV cell is effectively mitigated. At *C*_opt_ = 3, the temperature is only 40.5 °C, which is even lower in the case of the Ag mirror at *C*_opt_ = 1 and demonstrates a significant 55 °C lower temperature than that of the Ag mirror at *C*_opt_ = 3. This allows a possible 10-fold extended lifetime of the PV cell. The effective thermal management also benefits the photoelectric conversion, with the output power showing a positive correlation with *C*_opt_ within the test range. Notably, the output power from the PV cell in the asym-MDM HPT system has a 63.9% increment compared to the same PV cell in the standalone system at *C*_opt_ = 3.

Meanwhile, the solar power absorbed by Ni-based asym-MDM also enhanced the performance of the TEG, as shown in Figure 5c. A bare TEG usually has a poor absorption toward solar illumination (see Figure S6d for the absorption of a bare TEG), and therefore, the output electricity is limited even under high solar concentrations. When the Ni-based asym-MDM is attached to the hot side of the TEG, it serves as a better solar absorber, leading to a higher temperature on the hot side compared to a bare TEG. Thus, the asym-MDM-attached TEG will generate a larger temperature difference between the hot and cold sides of the TEG. As a result, the output power is significantly increased for all solar concentrations. At *C*_opt_ = 3, the asym-MDM-attached TEG generates 3.7 times more power than the bare TEG. Overall, the HPT system achieves huge improvements for both photoelectric conversion and thermoelectric generation.

The asym-MDM HPT system outperforms the current HPT systems employing spectrum-splitting techniques (e.g., dichroic mirror,^31^ DBR mirror,^12^ and nanofluid^32^) in several ways. First, asym-MDM consists of only three-layer subwavelength optical coatings, largely reducing the fabrication complexity and cost. As a comparison, both the dichroic mirror and DBR mirror rely on stacking multiple thin film layers with alternating refractive indices, with the thicknesses often in the range of micrometers and are at least an order of magnitude thicker than the asym-MDM. Second, the structural simplicity of the asym-MDM ensures its scalability for large-scale applications with mature optical coating techniques. In contrast, nanofluid-based selective absorbers face problems in large-scale production to maintain constant high-quality and homogeneous nanoparticles.^33^ Third, due to the absorptive nature, asym-MDM serves as the dual functionality of both a spectrum splitter and a solar absorber, while additional thermal receivers are required for dichroic mirror and DBR mirror HPT systems and thermal exchangers are necessary for nanofluid HPT systems.^34^ Moreover, asym-MDM can be designed to have ultralow iridescence by selecting appropriate dielectric material, while the dichroic mirror and DBR mirror are inherently highly iridescent,^35^ making them inappropriate for concentrated solar applications due to the mismatch between the reflection band and the external quantum efficiency of the solar cell under oblique incidence.

## 3 Conclusions

Our study presents a simple yet effective design of a dual-functional lithography-free asymmetric metal-dielectric-metal (asym-MDM) optical coating that serves both as a spectrum splitter and a thermal receiver in a solar hybrid photovoltaic/thermal (HPT) system. By strategically leveraging on the multiorder spectral responses of the asym-MDM, we realize a quad-band spectral response: a reflective photovoltaic band aligning with the external quantum efficiency of the amorphous Si solar cell, two thermal bands effectively absorbing the solar energy, and an infrared band exhibiting ultralow emissivity to suppress thermal radiation loss. Guided by the theoretical analysis, we explore the role of different metals as the top layer to realize better thermal management in the HPT system. We demonstrate that the Ni-based asym-MDM has the best photothermal conversion capability in harvesting the solar energy, while it can also split a narrowband of a solar spectrum to the solar cell that aligns with the peak of the external quantum efficiency of the solar cell. Furthermore, we introduce silica aerogel as a capping layer for the asym-MDM, effectively reducing convective heat loss and thus enhancing the solar-thermal performance. Eventually, we constructed a compact Ni-based asym-MDM HPT system, achieving 63.9 and 370% output power enhancements in the photovoltaic and photothermal modules, respectively. Our findings provide valuable insights into the development of efficient and cost-effective HPT systems, contributing to the advancement of solar energy harnessing and conversion technologies.

## 4 Methods

### Calculation of the Cavity Reflection Coefficient

Assuming that the incident electric field to the asymmetric MDM structure is *E*_0_, the total reflected electric field from the cavity of the MDM will then be a result of concurrent reflections given by the following:^24^
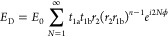
(5)where *t*_1a_ and *t*_1b_ are the transmission coefficients and *r*_1b_ and *r*_2_ are the reflection coefficients, as indicated in Figure 2a. The reflection coefficient from the cavity can then be obtained from *r*_D_ = *E*_D_/*E*_0_, which will give us eq (1) in the manuscript.

### Deposition of Asymmetric MDM

The three-layer asym-MDMs were deposited on 2 cm × 2 cm microscope glass slide substrates using an electron beam evaporation (EBE) system (Kurt J. Lesker PVD 75). All materials were in solid form and were placed in crucibles. In a typical deposition process, accelerated electrons transfered their energies to the materials, leading to evaporation of the materials and subsequent deposition onto the substrate. The deposition rates for the lossless dielectric TiO_2_ (73 nm) and bottom Ag (100 nm) layer were 0.5 and 1 Å s^–1^, respectively. For the three different top metal layers, deposition rates of 0.5, 0.5, and 0.1 Å s^–1^ were used for Ag (40.4 nm), Cu (35.5 nm), and Ni (8 nm), respectively.

### Numerical Calculation of the Reflection and Absorption Spectra

Numerical reflections for the asymmetric MDM were calculated using the transfer matrix method (TMM) written in MATLAB. Absorption is complementary to the calculated reflection and transmission, that is, *A* = 1 – *R* – *T*, and is complementary to the reflectance for opaque substrates.

### Optical Absorbance/Reflectance Measurements in the Ultraviolet–Visible–Near-Infrared and Mid-Infrared Regions

The hemispherical optical reflectance of the as-deposited asymmetric MDMs was measured in the spectral range of 0.25–2.5 μm using a PerkinElmer Lambda-900 double-beam spectrophotometer coupled with a 50 mm diameter integrating sphere. Similarly, hemispherical reflectance in the mid-infrared region (2.5–25 μm) was measured using a Thermo Fisher Scientific Nicolet 6700 FTIR spectrometer coupled with a PIKE research integrating sphere. As the MDM is opaque, the absorbance is complementary to the measured reflectance in the ultraviolet–visible–near-infrared and mid-IR range; therefore, absorbance is obtained using *A* = 1 – *R*. For the visible reflection at oblique angles, we employed a self-built experimental setup, as shown in Figure S4c. Light from a halogen white light source (Thorlabs: SLS301, wavelength range: 360–2700 nm) was first collimated by the combination of an iris and an objective lens, whose polarization was then selected by a broadband Glan–Thompson polarizer (Melles Griot: 03 PTH 112/C, wavelength range: 350–2300 nm). Eventually, the reflected beam from the FROC went through another broadband Glan–Thompson polarizer and was coupled into the fiber collector of the spectrometer (Photon Control: SPM001, wavelength range: 300–1000 nm) by an objective lens. The measured reflectance of the asymmetric MDM was normalized to the reflectance of a Ag mirror.

### Photovoltaic Measurements

A solar simulator (Sanyou) with an AM1.5G air mass filter was first calibrated for 1 sun (1000 W m^–2^) using an NREL-certified PV reference solar cell. The measured power of a thermopile power meter (FieldMax II TO, Coherent; minimum measurable power ±10 μW) was set to a wavelength of 500 nm, corresponding to 1000 W m^–2^ from the calibrated solar simulator, and was used as a unit of one optical concentration. Two Fresnel lenses were consecutively mounted at the output port of the solar simulator to enhance the optical concentration. The simulator current was varied to adjust solar irradiance from 1000 to 3000 W m^–2^ at the plane of the PV cell. The single-junction amorphous Si PV cell was purchased and cut, and two wires were soldered to create a functioning PV cell. The temperature was measured using thermocouples, and we have reported the equilibrium temperature. Power–voltage curves were obtained using a Keithley 2450 source meter by sweeping the voltage in a certain range, and maximum powers were found at the peak of the curve.

## Data Availability

The data that support the findings of this study are available from the corresponding authors upon reasonable request.

## Supporting Information Available

The following Supporting Information is available free of charge at the ACS Web site. The Supporting Information is available free of charge at https://pubs.acs.org/doi/10.1021/acsphotonics.4c01315.

Additional details of simulation, material properties, and experimental measurements on the asym-MDM; photograph of asym-MDMs with different top metal layers and experimental setups; additional discussion on how the top layer material and its thickness affect the spectral response; and analysis on the iridescence of asym-MDMs (PDF)
