# Supplementary material for: High-Efficiency Solar Hybrid Photovoltaic/Thermal System Enabled by Ultrathin Asymmetric Fabry–Perot Cavity
Source: ACS Photonics. 2025 Feb 6;12(2):628–35. doi: 10.1021/acsphotonics.4c01315 (PMC11843714; doi:10.1021/acsphotonics.4c01315)
Supplement: Supplementary file 1 — ph4c01315_si_001.pdf [file ph4c01315_si_001.pdf]

Supporting Information

High-Efficiency Solar Hybrid Photovoltaic/Thermal System enabled by Ultrathin Asymmetric Fabry-Perot Cavity

*Ran Wei, Tianshu Xu, and Chunlei Guo**

The Institute of Optics, University of Rochester, Rochester, NY 14627, USA

* *chunlei.guo@rochester.edu*

Supporting information: Figure S1-S10, with additional simulation and experimental details, materials, discussions, and photographs of experimental setup.

Supporting Note 1: Choice of different top layer material and thickness on the bandwidth of spectral response.

Supporting Note 2: Iridescence of asym-MDM vs. refractive index of the lossless dielectric material.

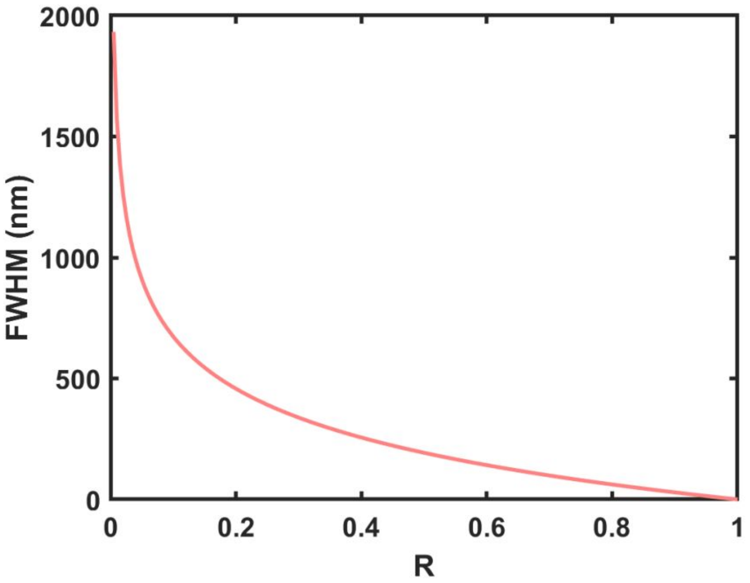

Figure S1. Full width at half maximum (FWHM) calculated as a function of the reflectance of the top metal layer according to Equation (2) in the manuscript. Here, λ, n_d, and L are taken to be 800 nm, 2.52 (the refractive index of TiO_2 at 800nm), and 73nm, respectively.

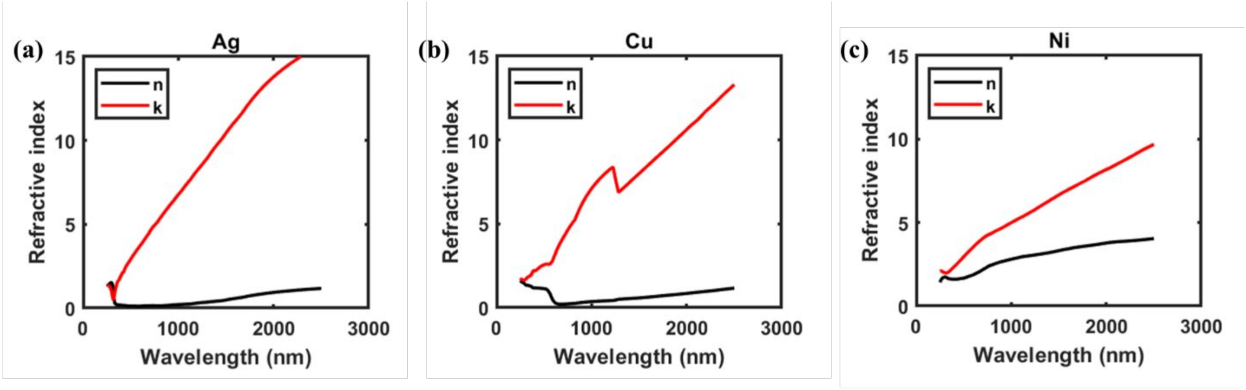

Figure S2. Complex refractive indices for the three different materials involved in the manuscript for obtaining the numerical calculation results.

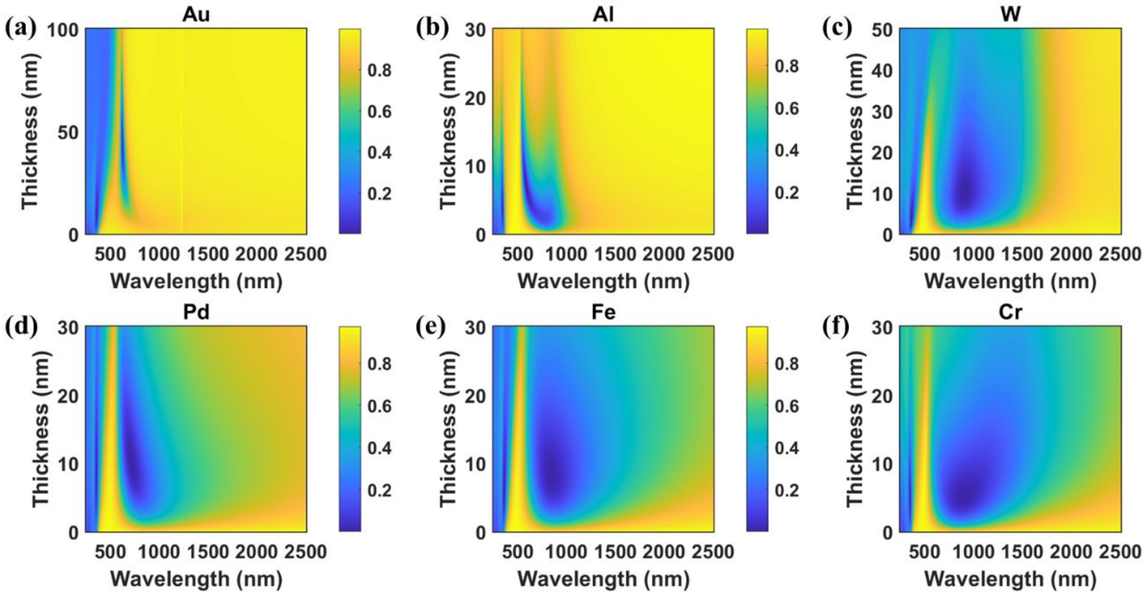

Figure S3. Numerically calculated reflectance of more top metal materials.

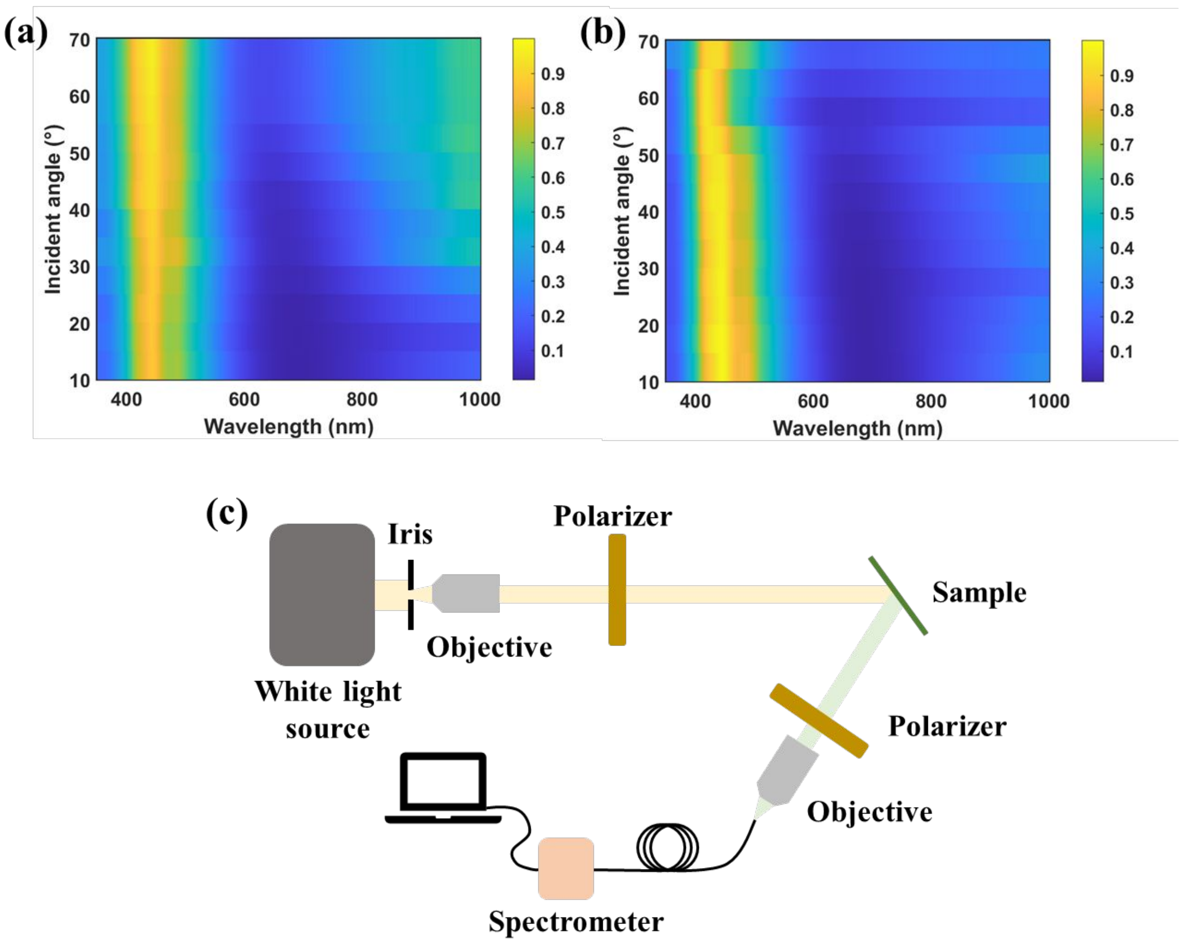

Figure S4. Experimentally measured angular reflectance of the Ni-based a-MDM under s-polarized illumination **(a)** and unpolarized illumination **(b)**. **(c)** Schematics of the experimental setup used for measuring angular reflectance under different polarization.

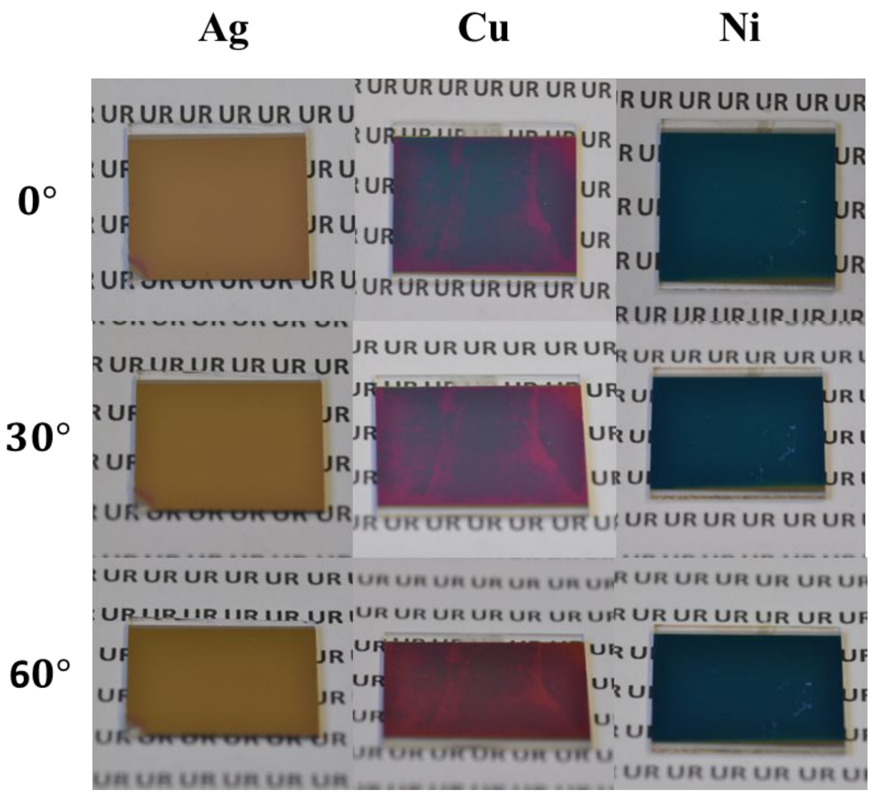

Figure S5. Photos of the three deposited asym-MDMs taken at different angles, where the surface colors of all deposited asym-MDMs remained unchanged with different observation angles.

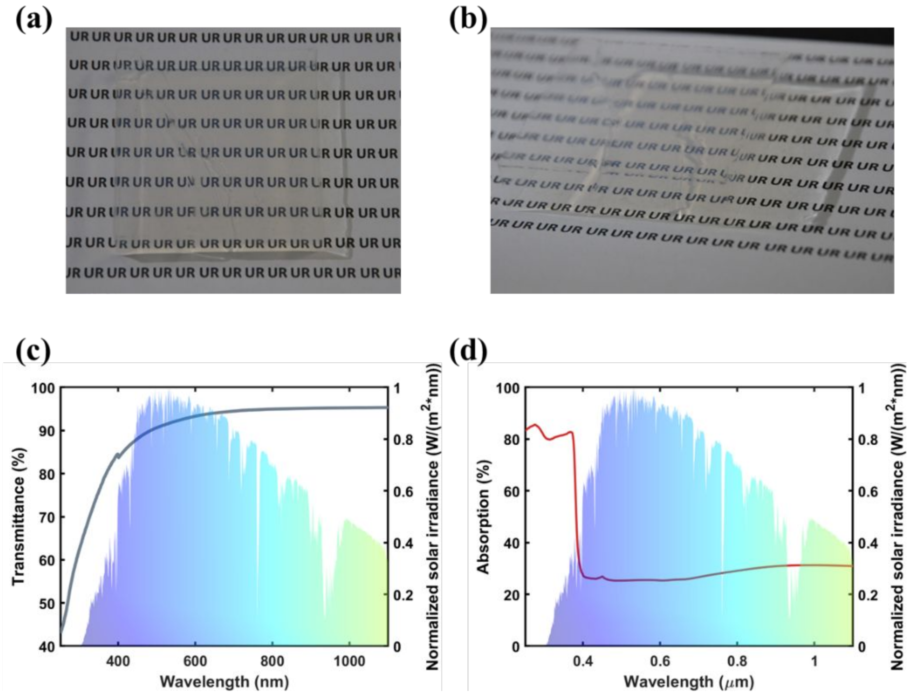

Figure S6. (a-b) Appearance of the silica aerogel used in the experiment. **(c)** Transmittance of the silica aerogel. **(d)** Absorption of the TEG.

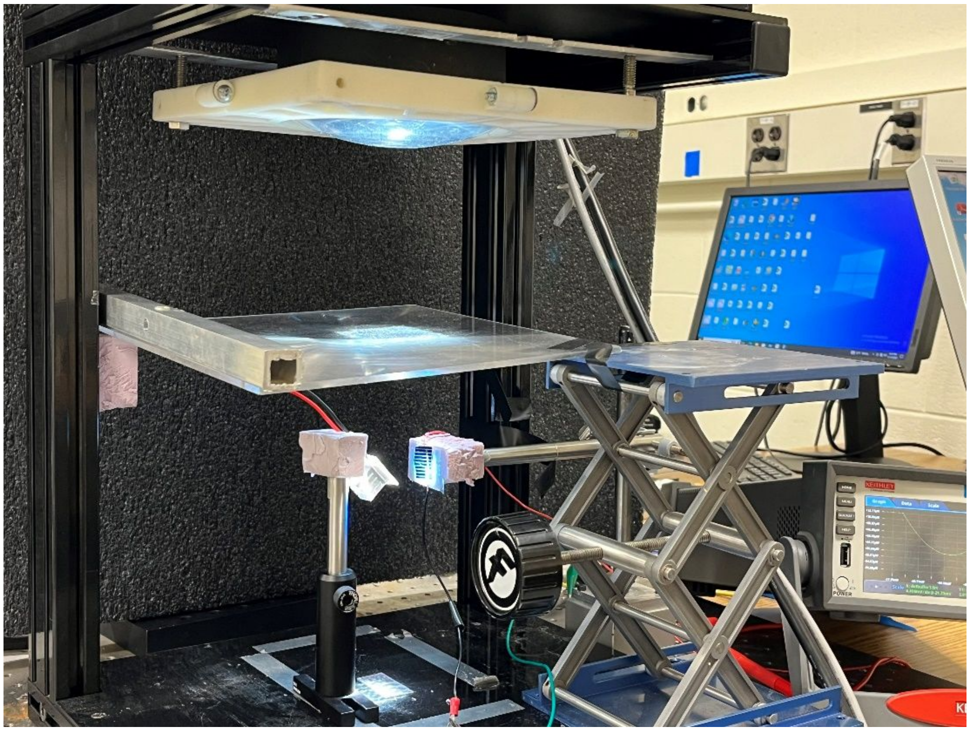

Figure S7. Photo of the experimental setup for testing the asym-MDM HPT system. Light from the solar simulator is concentrated by two lenses and incidents onto the asym-MDM, where the PV band is reflected to the truncated amorphous Si PV cell. A TEG is attached to the back of the asym-MDM using high-temperature thermal paste. The power output from the PV cell and the TEG are monitored by a source meter.

Supporting Note 1: Choice of different top layer material and thickness on the bandwidth of spectral response.

Following Fresnel's law, the reflection coefficient at the air-metal interface is determined by $\rho = (n_a - n_m)/(n_a + n_m)$, where n_a represents the refractive index of air and is assumed to be 1, and $n_m = n + ik$ is the complex refractive index of metal. For noble metals with $n \ll k$, the reflection coefficient can be approximated as $\rho = -n_m/n_m = -1$, corresponding to unity reflectance of $R = |\rho|^2 = 1$. However, for lossy metals, the real part of ρ cannot be neglected, resulting in lower reflectance, as illustrated in **Figure S8(a)**. Furthermore, our observations from Figure 2 demonstrate that the bandwidth of absorption peaks narrows with an increase in the thickness of the metal layer. As we have already mentioned in the manuscript, the full width at half maximum (FWHM) of an Fabry-Perot (FP) cavity at normal incidence is given by,

$$\delta\lambda = \frac{\lambda^2(1 - \sqrt{R})}{2\pi n_d L R^{0.25}} (S1)$$

where λ denotes the resonant wavelength, R signifies the reflectance, and n_d and L represent the refractive index and physical thickness of the lossless dielectric layer, respectively. Apparently, larger R values correspond to narrower FWHM. Accordingly, we calculated the reflectance of thin metal films atop a lossless dielectric substrate of TiO_2 at λ=800 nm for various thicknesses, as shown in **Figure S8(b)**. All metal thin films exhibit a similar trend, with reflectance initially increasing with thickness and eventually approaching a constant value. Notably, for metals whose refractive indices have imaginary parts much larger than the real parts (e.g., Ag, Au, and Cu), they display almost identical responses, converging to ~100% reflectance at a certain thickness. In contrast, metals with comparable imaginary and real parts of refractive indices (e.g., Ni, Pt, and Ti) exhibit comparatively lower reflectance, leading to broadened absorption bands. **Figure S8(c)** further presents a complete picture of how the reflectance (R in Equation (S1) changes with n, k, and thickness of the metal of interest.

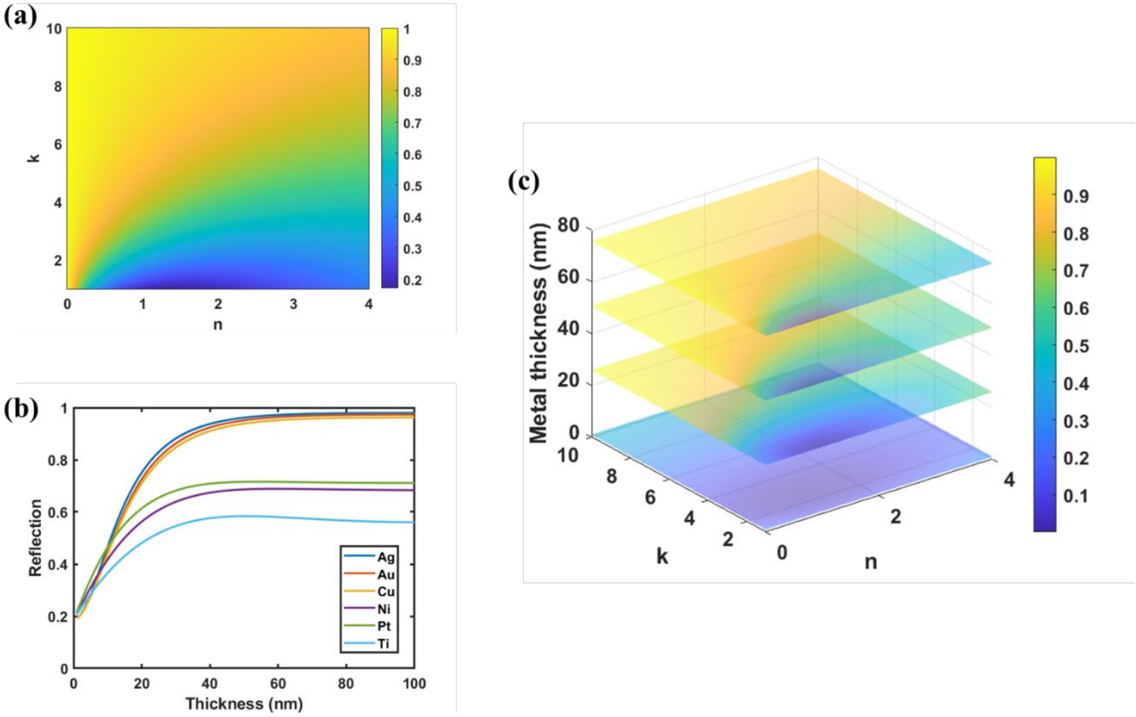

Figure S8. (a) Reflectance at air-metal interface with respect to different real (n) and imaginary (k) parts of metal. **(b)** Reflectance at the air-metal interface with varying thicknesses of different types of metals, where the first three has refractive indices with imaginary part much larger than the real part, and the latter three has refractive indices with imaginary part similar to the real part. **(c)** Reflectance of a metal thin film on a $TiO2$ dielectric layer as a function of n, k, and thickness of the metal layer at λ=800 nm.

Supporting Note 2: Iridescence of asym-MDM vs. refractive index of the lossless dielectric material.

For an asym-MDM, at the resonant wavelength under normal incidence, the light reflected from the top layer destructively interferes with the light reflected from the cavity. This requires a π phase difference between the two interfering beams. Since there will be an additional π phase shift due to the hard reflection at the top surface, the phase accumulation within the cavity therefore should be a multiple of 2π. Assuming the refractive index and thickness of the cavity are n and t, respectively, the phase difference between the two interfering beams can thus be given by,

$$\phi_1 = \frac{4\pi nt}{\lambda_0} - (S2)$$

where λ_0 is the resonant wavelength at the normal incidence. In the case of oblique incidence with an angle θ, the phase difference between the two reflecting beams will change and can be expressed as,

$$\phi_2 = \frac{4\pi ntcos\theta'}{\lambda_0} - \pi = \frac{4\pi t\sqrt{n^2 - \sin^2\theta}}{\lambda_0} - (S3)$$

Here, θ' is the angle of the refracted beam within the cavity and is related with the incident angle by $sin\theta = nsin\theta'$. The iridescence then comes from the difference between ϕ_1 and ϕ_2,

$$\Delta\phi = \phi_1 - \phi_2 = \frac{4\pi t}{\lambda_0}\left(n - \sqrt{n^2 - \sin^2\theta}\right)(S4)$$

Notice $\sin^2\theta \geq 0$, therefore ϕ_1 at the normal incidence will always be larger than ϕ_2 at the oblique incidence. Subsequently, the resonant wavelength will witness a blue shift to compensate for the inadequate phase difference. **Figures S9(a)** and **S9(b)** numerically calculate $\Delta\phi$ and resonant wavelength with different incident angles for an asym-MDM according to Equation (S4) with low and high refractive indices, respectively. Both cavities have the same optical thicknesses with resonant wavelength at 800 nm under normal incidence. At high refractive index, the asym-MDM experiences a much smaller change in $\Delta\phi$ and exhibits significantly lower iridescence, i.e., the resonant wavelength does not change much with incident angle, while the resonant wavelength rapidly shifts when the cavity has a low refractive index. This analysis assumes that the dielectric material is dispersion-less for simplicity. However, the general behavior still holds when introducing typical dispersion to the cavity material (**Figure S10**). Therefore, by tuning the refractive index within the cavity through appropriate selection of material, the iridescence can be flexibly controlled.

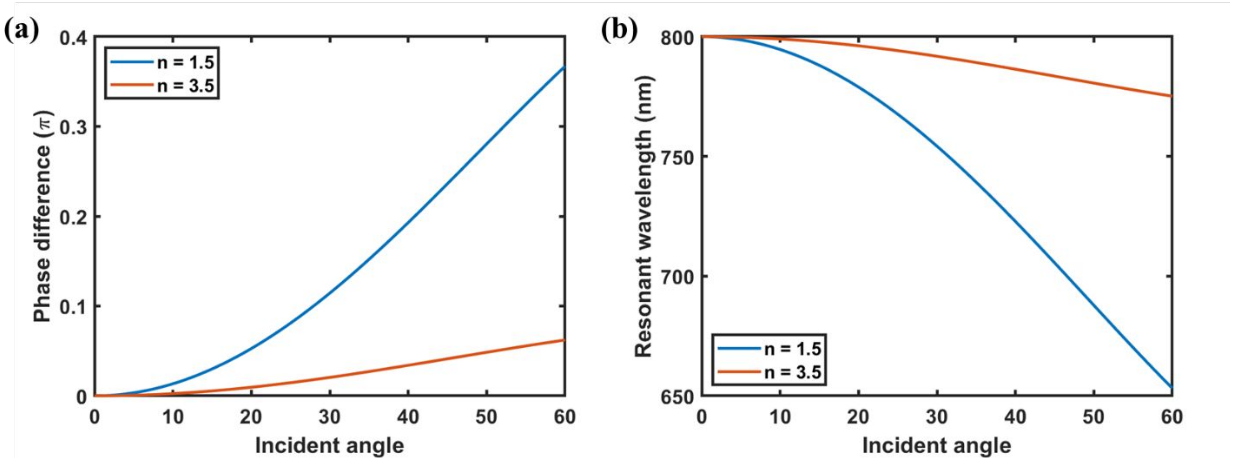

Figure S9. Theoretical analysis on iridescence of asym-MDM with the refractive index of the dielectric material. **(a)** Phase differences calculated according to Equation (S4) for low and high refractive indices of dielectric material, respectively. **(b)** Shift of resonant wavelengths for asym-MDMs with low and high refractive indices of dielectric material, respectively.

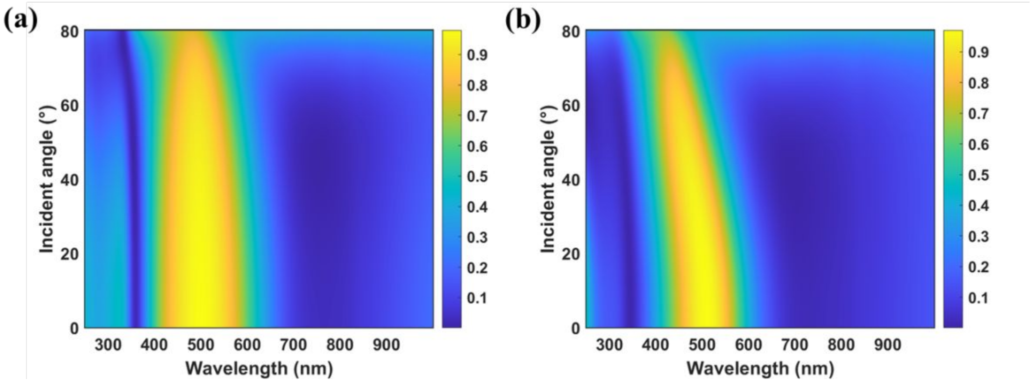

Figure S10. Angular reflectance for p-polarized light of asym-MDMs incorporating **(a)** high index material TiO_2 (73 nm) and **(b)** low index material MgF_2 (150 nm) as the dielectric, respectively. Both asym-MDMs have a Ni top layer (8 nm) and an Ag bottom layer (100 nm). The former asym-MDM exhibits significantly lower iridescence than the latter one.
